# Confound, Cause, or Cure: The Effect of Cannabinoids on HIV-Associated Neurological Sequelae

**DOI:** 10.3390/v13071242

**Published:** 2021-06-26

**Authors:** Alexander Starr, Kelly L. Jordan-Sciutto, Eugene Mironets

**Affiliations:** Department of Basic and Translational Sciences, University of Pennsylvania, Philadelphia, PA 19104, USA; alexander.starr@pennmedicine.upenn.edu (A.S.); eugene.mironets@gmail.com (E.M.)

**Keywords:** cannabinoids, HIV, HAND, CB_1_, CB_2_, antiretroviral

## Abstract

The persistence of human immunodeficiency virus-1 (HIV)-associated neurocognitive disorders (HAND) in the era of effective antiretroviral therapy suggests that modern HIV neuropathogenesis is driven, at least in part, by mechanisms distinct from the viral life cycle. Identifying more subtle mechanisms is complicated by frequent comorbidities in HIV^+^ populations. One of the common confounds is substance abuse, with cannabis being the most frequently used psychoactive substance among people living with HIV. The psychoactive effects of cannabis use can themselves mimic, and perhaps magnify, the cognitive deficits observed in HAND; however, the neuromodulatory and anti-inflammatory properties of cannabinoids may counter HIV-induced excitotoxicity and neuroinflammation. Here, we review our understanding of the cross talk between HIV and cannabinoids in the central nervous system by exploring both clinical observations and evidence from preclinical in vivo and in vitro models. Additionally, we comment on recent advances in human, multi-cell in vitro systems that allow for more translatable, mechanistic studies of the relationship between cannabinoid pharmacology and this uniquely human virus.

## 1. Introduction

There are an estimated 38 million people living with human immunodeficiency virus-1 (HIV) globally, of which only 81% are likely aware of their HIV status and only 67% are being treated with antiretroviral therapy (ART) [1]. While great strides have been made in the prevention, diagnosis, and treatment of HIV infection, it continues at epidemic levels. Global efforts continue to focus on preventing new infections and reducing viral transmission with effective ART, yet there remains an immense burden of care, not simply for viral control, but also for treating the long-term consequences of infection and treatment, including immune system dysfunction, gastrointestinal damage and anorexia, neuropathic pain, and HIV-associated neurocognitive disorders (HAND).

HAND is a descriptor for a number of mood, memory and learning, cognitive, and motor disorders associated with central nervous system (CNS) damage following HIV infection. HAND can be classified into three subcategories based on severity: (1) asymptomatic neurocognitive impairment (ANI), which does not obviously affect everyday life but is detectable using formal cognitive tests; (2) mild neurocognitive disfunction (MND), with symptomatic consequences that impair normal function; and (3) HIV-associated dementia (HAD) [2]. Prior to ART, more severe forms of HAND were frequently associated with HIV encephalitis (HIVE), in which HIV itself and/or opportunistic infections introduced by acquired immune deficiency syndrome (AIDS) caused swelling and gross histological damage in the brain. In the ART era, effective treatment significantly reduces the plasma viral load, restores immune function, extends the life span to near normal, and reduces the frequency of HIVE and HAD; however, the frequency of HAND remains near 50% [3,4,5].

The fact that HAND persists despite viral suppression suggests that evaluating factors secondary to basic HIV biology might reveal alternative or synergistic causative mechanisms and/or opportunities for intervention. Among these factors, people living with HIV (PLWH) are most frequently exposed to (1) ART and (2) psychoactive drugs of abuse (DoA) that are known to cause neuronal damage and impair cognitive function. While only a few ARTs have been clinically associated with peripheral neuropathies, neurodevelopmental defects, and cognitive impairment [6], considerable laboratory literature demonstrates ART neurotoxicity in vivo in animal models and in vitro in animal and human neuronal models [7,8]. Putative mechanisms for ART toxicity include excitotoxic glutamate and proinflammatory cytokine release from ART-treated macrophages, microglia, and astrocytes; impaired oligodendrocyte differentiation due to lysosomal disfunction; and direct neuronal toxicity mediated by oxidative damage and activation of the integrated stress response [9,10,11]. The developing field of ART neurotoxicity has been reviewed extensively [12], reflecting scientific intrigue regarding that which can both cure and cause HIV-associated pathologies.

The paradoxical nature of ART toxicity is reflected in the pharmacology of the compounds reviewed here, cannabinoids. Cannabis use within the HIV community is highly prevalent [13,14] and should be considered when conducting studies that aim to understand HIV pathogenesis. To date, preclinical research on the effect of cannabinoids is limited, despite widespread use for recreational and medical purposes [15,16,17]. Traditionally considered a psychoactive DoA in PLWH, both endogenous and exogenous cannabinoids may function as therapeutic modulators of the immune system and neuronal signaling in HAND. This review will briefly summarize current knowledge regarding HAND pathogenesis and the components of the human endocannabinoid (endoCB) system, before exploring the currently available research surrounding clinical and pre-clinical data on the interplay of cannabinoids, HIV infection, and CNS sequelae. By organizing clinical and pre-clinical data according to phenotypes observable both in patients and in models (e.g., HIV replication dynamics, inflammatory cytokine release, blood–brain barrier integrity), we hope to highlight parallels between patient outcomes and related cellular and molecular mechanisms. We will also examine how emerging iPSC technology in human CNS modeling can be used to better translate benchtop revelations to bedside interventions.

### 1.1. HIV Infection in the CNS and Neurotoxic Sequelae

HIV is thought to enter the brain either as a free virion or via infiltration of infected peripheral cells that traverse the blood–brain barrier (BBB) into the parenchyma of the central nervous system (CNS). Genetic analyses of HIV samples from the blood and CSF show compartmentalization (distinct genetic signatures reflecting independent viral replication in isolated cell types) within months of initial infection [18]. Several genetic studies, recently reviewed by Spudich et al., suggest that early compartmentalized sequences are T cell tropic; however, over time, and especially with prolonged ART, macrophage tropic sequences become more prevalent [19]. Evidence that HIV-infected monocytes can cross the blood–brain barrier, as reviewed by Williams et al., suggests that peripheral monocytes and their differentiated macrophages may contribute to the establishment of the CNS viral reservoir [20]. Further study is needed to determine the relative contributions of different peripheral immune cell types to HIV entry into the CNS. After transmigration has occurred, immunohistochemical studies have identified HIV protein, DNA, and RNA in brain-resident macrophages, microglia [21], and astrocytes [22]; however, neurons and oligodendrocytes are not infected. Infected cells have the capacity to secrete inflammatory chemokines and cytokines, driving neighboring, uninfected brain-resident macrophages, microglia, and astrocytes into a proinflammatory state. The resulting environment, which contains excess glutamate [23,24], reactive oxygen species [25,26], and proinflammatory cytokines, leads to excitotoxic neuronal damage and dysfunction. HIV also damages the BBB, allowing for inappropriate ingress of peripheral cells and opportunistic microbes, creating a feed-forward loop of HIV infiltration and inflammation. Importantly, these sequelae of HIV infiltration into the CNS were originally characterized in the context of pre-ART encephalitis. While inflammation, white matter disruption, and neuronal dysfunction persist in the ART era, it is likely that specific mechanisms of viral persistence, inflammation, and neurotoxicity, along with the morphological and molecular markers of those mechanisms, differ in an aging, virally suppressed population [27]. Intriguingly, cannabinoids target receptors integral to the regulation of both excitatory neuronal signaling and inflammation, providing an opportunity for cross talk between the most robust neurotoxic consequences of HIV infection and the most commonly used DoA in HIV^+^ populations.

### 1.2. The Endocannabinoid System and Cannabinoid Pharmacology

The human endogenous cannabinoid system consists of the cannabinoids 2-arachidonoylglycerol (2-AG) and anandamide (AEA); their synthesis enzymes, diacylglycerol lipase (DGL, *DAGLA*) and N-acyl phosphatidylethanolamine phospholipase-D (NAPE-PLD, *NAPEPLD*); their hydrolytic enzymes, monoacylglycerol lipase (MAGL, *MGLL*) and fatty acid amide hydrolase (FAAH, *FAAH*); and their Gi/o-coupled receptors, cannabinoid receptor type 1 (CB_1_, *CNR1*), CB_2_ (*CNR2*), and the less well-described orphan G protein-coupled receptors 55 (GPR55, *GPR55*) and GPR18 (*GPR18*). Additional putative endoCBs and receptors have been identified; however, their functions in the endoCB system, especially in the context of neuroHIV, have not been well established, and they will not be covered in this review [28]. CB_1_ is primarily expressed in the CNS in neurons, where its presynaptic activation inhibits neurotransmitter release. CB_2_ is found primarily in peripheral immune cells; however, in the brain, it is robustly expressed in endothelial cells, brain-resident macrophages, and microglia. CB_2_ activation is immunomodulatory, inhibiting activation and proinflammatory cytokine release [28,29].

*Cannabis sativa*, or marijuana, contains a number of phytocannabinoids, plant derived compounds that act on endoCB receptors [30]. The relative concentrations of cannabinoids can vary greatly between plants, and certain plants are specifically cultivated for their cannabinoid profiles. Generally, Δ9-THC, an agonist for both CB_1_ and CB_2_, with higher CB_1_ affinity, makes up approximately 17% of all cannabinoid compounds in naturally occurring cannabis. Δ9-THC inhibits neurotransmitter release, resulting in the psychoactive effects associated with cannabis use and abuse. The pharmacology of cannabidiol (CBD), which comprises approximately 9% of all cannabinoids, is more complex, with apparent antagonistic effects at both CB receptors well below the binding affinity [31]. 

Several synthetic cannabinoids have been developed, with varying degrees of receptor specificity, affinity, potencies, and signaling biases [32]. In addition to specific and non-specific agonists, specific inverse agonists, as opposed to true antagonists, are frequently used as such to determine receptor dependence in experimental investigations. In clinical observations and treatment, phytocannabinoids are the most frequently encountered compounds, while bench researchers frequently take advantage of synthetic cannabinoids to ensure consistent, receptor-specific signaling.

### 1.3. Method of Literature Review

Literature databases, including Pubmed and Google Scholar, were searched for key words, including “Cannabis,” “Cannabinoids,” “Marijuana,” “Endocannabinoid,” “CB_1_,” or “CB_2_” combined with “HIV,” “HIV-Associated Neurocognitive Disorders,” “HAND,” “HIV Brain,” and “HIV Neuron.” Article selection focused on novel research and clinical studies, although previous reviews on related topics were also selected. Clinical and laboratory studies that solely focused on HIV in peripheral tissues, such as gut or lung, were excluded. Articles were selected from the year 1990 to the present, with emphasis placed on studies in the post-ART era. If an author appeared in more than two articles collected from the general subject search, the author’s name was specifically searched to collect all relevant work.

## 2. Cannabinoids × HIV: Prevalence, Cross Talk, and Clinical Outcomes

### 2.1. Purpose and Prevalence of Exocannabinoid Use in PLWH

Cannabis use is common among PLWH. While the observed incidence varies and often relies on self-reporting, common frequencies in the general HIV population range from 11.7% to 37.3% [33,34,35,36,37,38,39,40,41,42] across multiple international cohorts. Importantly, these numbers best approximate lifetime cannabis use in PLWH, since data on current use during HIV treatment are not always available. The most cited reasons for use include management of neuropathic pain (approximately 39.4%), relief of anxiety and depression symptoms, and improving appetite [33,34,43]. Hartzler et al. identify approximately half of cannabis-using PLWH as being at risk for cannabis use disorder [44]; however, not all cannabis use in PLWH is independently self-administered.

HIV is one of the few diseases where therapeutic cannabinoids are approved by the US Food and Drug Administration and prescribed in the form of synthetic, orally administered THC. This compound, dronabinol, along with smoked cannabis, is effective at stimulating appetite to combat HIV-associated anorexia [45] and improve weight gain [46]. This is likely accomplished by stimulation of the appetite hormones ghrelin and leptin [47]. However, a more recent Cochrane systematic review indicated that longer studies would be necessary to truly evaluate the consistency and significance of dronabinol’s reported benefits [48].

Another clinically reported indication for cannabinoids in HIV is pain management. A number of clinical trials show that smoking cannabis is effective for relief of HIV-associated neuropathic pain [49,50], and a meta-analysis of such studies concluded that smoked cannabis is effective for this purpose [51]. In addition, cannabis use among PLWH with chronic pain reduced the odds of prescription opioid analgesics, indicating it acted as an alternative analgesic therapy [43]. This effect is mediated by specific phytocannabinoids, as cannabidivarin, a non-psychoactive phytocannabinoid, failed to alleviate HIV-associated neuropathic pain [52].

Studies note that cannabis use predisposes PLWH to dangerous behavior, including abuse of alcohol and other psychoactive substances. Cannabis use was positively correlated with increased alcohol consumption in an HIV^+^ cohort, and in HIV^+^ Russian risky drinkers, cannabis increased dangerous drug- and sex-related behaviors [53,54]. However, HIV^+^ men who use only cannabis are less likely to contract additional sexually transmitted infections than are non-drug users or poly-drug users, so increased risky sex behavior may not predict additional harm [55]. Most importantly, cannabis use has not been associated with mortality or progression of HIV infection to AIDS, suggesting that cannabinoids are generally well tolerated among PLWH, relative to the general population [56,57]. Following these observations, and to better understand the interaction between HIV and cannabinoids, researchers have examined cellular and molecular biology beyond overt clinical symptoms and gross mortality.

### 2.2. Effect of HIV Infection on the Endocannabinoid System

Clinical manifestations of the interplay between cannabinoids and HIV begin with observations of co-regulation between the two stimuli. HIV infection alters the expression and function of multiple components of the endoCB system, even in the absence of exocannabinoid exposure. Immunohistochemical analysis of HIVE brain tissue revealed increased expression of CB_1_ in neurons and white-matter-resident microglia and increased CB_2_ across microglia, perivascular macrophages, and astrocytes [58]. While no other studies have confirmed or expanded upon this discovery in HIV^+^ human samples, experiments in SIV-infected rhesus macaques provide a closely correlated clinical model. SIV infection also increases expression of CB_2_ in microglia, brain-resident macrophages, and T cells [59]. Intriguingly, FAAH was found to be increased in astrocytes, indicating that the increased systemic sensitivity to cannabinoids due to receptor upregulation might be modulated by more rapid degradation of cannabinoids [59]. In vivo models have also shed light on the effects of exocannabinoids on the endoCB system in the context of SIV infection. Infected macaques developed tolerance to the behavioral alterations associated with chronic Δ9-THC exposure, and follow-up post-mortem analysis found decreased expression of both CB_1_ and CB_2_ in the hippocampus [60]. While these studies lay the groundwork for understanding how HIV infection alters the endoCB system in the CNS, further analyses in cohorts of ART-treated individuals with and without HAND are necessary to understand these phenomena in a current, clinically relevant context. Nonetheless, the fact that HIV infection modifies the endoCB system suggests that the expected effects of cannabinoid exposure, be it from cannabis abuse or a prescribed therapeutic, may be different in HIV^+^ populations. This will be further explored in a discussion of the preclinical data demonstrating the effects of HIV infection on the endoCB system.

### 2.3. Effect of Cannabinoids on HIV Infection Dynamics and Treatment Adherence

Just as HIV infection can alter the endoCB system, cannabinoids can alter the dynamics of HIV infection. This can be explained by two key factors: biological interplay between cannabinoids and the viral replication process and/or cannabis-associated behavioral alterations that influence adherence to HIV treatment. Both mechanisms have been studied in multiple HIV^+^ cohorts, with some conflicting findings. HIV^+^, ART-treated cannabis users display equal or lower viral load and circulating HIV nucleic acids than non-users [15,61,62], especially in the context of poly-drug use [63,64]. This is supported by observations in macaques infected with SIV [60,65,66]. In addition, a meta-analysis of clinical studies in African cohorts of men who have sex with men suggested that cannabis use was associated with a lower risk of acquiring HIV infection [35]. While these findings are countered by evidence of higher levels of HIV RNA in semen and increased risk of detectable viral load at clinic visits in HIV^+^ cannabis users [67,68], the majority of clinical and in vivo evidence suggests cannabis use suppresses markers of viral replication.

In addition to biological interactions driving this observed viral suppression, the behavioral effects of psychoactive cannabis abuse may result in altered adherence to HIV treatment, including care appointments and medications. Kipp et al. report that daily marijuana use is associated with failed clinic attendance, and Bonn-Miller et al. report poor ART adherence in cannabis-dependent PLWH [69,70]. However, several other studies suggest cannabis use is not associated with altered ART fidelity [71,72,73,74,75,76]; thus, evidence of altered viral dynamics is not likely associated with altered ART consumption. One caveat for these studies is frequent use of other drugs of abuse in HIV^+^ cohorts. Socias and Milloy performed a meta-analysis of studies examining drugs of abuse and ART adherence, finding that cannabis was the sole DoA not associated with impaired ART adherence [77]. Other important factors to consider when comparing and conducting these studies is frequency of cannabis use, duration of cannabis use, and recency of cannabis use prior to data collection. Further stratification of cohorts may resolve contradicting conclusions regarding ART adherence and, ultimately, all other measures of cannabinoid x HIV associations. 

Importantly, exocannabinoids are processed by—and alter the function of—cytochrome P450 and other key drug metabolism enzymes and transporters [78], which can impact ART efficacy irrespective of adherence. While prescribed cannabinoid use does not significantly impact the pharmacokinetics of the HIV protease inhibitors indinavir or nelfinavir [79], further pharmacokinetic studies across ART classes would expand our knowledge of these potentially critical interactions.

### 2.4. Effect of Cannabinoids on Central and Peripheral Inflammation and Immunity in HIV

Beyond regulation of HIV infection dynamics, clinical observations indicate that cannabinoids can have a major impact on the primary target of HIV, the immune system. Cannabis use is linked with increased CD4^+^ and CD8^+^ T cell counts among multiple HIV cohorts [15,80] and fewer immune cells expressing CD16^+^ and other markers of activation [81,82]. In addition, levels of both plasma and CSF proinflammatory cytokines and soluble receptors are reduced in cannabis users, including IL-16, TNFRII, IL-23, TNF-α, and IP-10 [81,82,83,84]. Reduced monocyte activation markers have also been observed in the context of poly-drug abuse of cannabis and methamphetamine in PLWH [85]. Combined, these studies suggest that cannabinoids continue to exert their classical anti-inflammatory effects on monocyte lineage cells in the context of HIV infection, while protecting T lymphocytes from HIV-associated toxicity.

Cannabis use is associated with protective anti-inflammatory effects in the context of HIV and hepatitis C virus (HCV) co-infection. Regular cannabis use correlates with reduced steatosis, mortality, and insulin resistance in co-infected individuals [86,87,88] and is not associated with progression of liver fibrosis [89]. Importantly, cannabis use showed no observable effect on HIV RNA, viral load, or the circulating CD4^+^ cell count in HIV/HCV co-infection [90,91], suggesting that the protective effect of cannabinoids in this context is not mediated by altered HIV replication. Supporting the role of cannabinoids as modifiers of HIV/HCV pathology, a variant of cannabinoid receptor 2, 63 RR, is associated with more severe necroinflammation in co-infected individuals [92]. The same variant was connected with a higher rate of HIV acquisition among HCV^+^ individuals [93]. Further studies of this variant and its role in HIV neuropathology may offer better stratification of cannabinoid effects both in prescribed use and in marijuana users.

### 2.5. Cannabinoids as a Modifier for HAND Risk

Ultimately, several factors combine to modify the risk of HAND frequency, progression, and symptomology, including cannabinoid prescription and abuse, virally induced endoCB system alteration, disrupted HIV replication, and cannabinoid immunomodulation. Clinical measures of neurocognitive performance in PLWH who use cannabis have produced contradicting results, with evidence suggesting that recency and frequency of use affects cognitive performance. Lorkiewicz et al. observed that current marijuana use predicts reduced cognitive function but past use does not [94]. Contrastingly, early-onset use was correlated with impaired learning and memory, while late-onset use was not in a study by Skalski et al. [95]. In a Dutch cohort, varied frequencies of cannabis use were negatively associated with cognitive performance [96], while Thames et al. reported increased cognitive impairment with heavy use but improved verbal fluency with light use [15]. More recent studies, especially in older HIV^+^ populations, find a history of cannabis use is associated with improved cognitive performance, including processing speed, visual learning and memory, motor abilities, verbal fluency, and learning [97,98]. A report stating that lifetime cannabis use increased the likelihood of “SuperAging”—exceptional cognitive performance in older adults—among PLWH corroborates a potential long-term benefit of cannabis use in HAND [99]. The variety of published data suggests that studies of neurocognitive disfunction in HIV^+^ cannabis users must report and control for specific circumstances of cannabis consumption and highlights the possibility of differential effects of cannabis on specific cognitive tasks.

In addition to functional readouts of cognition, a number of imaging studies reveal the effects of cannabis use on the gross structure of the HIV-infected brain. Functional MRI-based brain network analysis revealed abnormal connectivity in HIV^+^ individuals; however, these alterations were not found in PLWH who use cannabis [100]. Marijuana use in PLWH is also associated with increased mean diffusivity in the right globus pallidus, but this does not correlate with impaired cognitive function [101]. Other brain structural abnormalities are associated independently with either cannabis consumption or HIV infection, but they do not show any combinatorial effect [16,101]. In ART-treated PLWH, increased CD4^+^ T cell recovery is associated with increased abnormalities in white matter and subcortical gray matter volumes, suggesting that recovery of immune function may be associated with damaging neuroinflammation [102]. Suppression of this immune activation may explain the structural protection observed in cannabis users in the studies above.

A primary suspected cause of HAND is a persistent viral reservoir in the brain, leading to chronic activation of brain-resident immune cells. Indeed, PET imaging of labeled microglia showed that the number of activated microglia is inversely correlated with cognitive performance in otherwise virally suppressed PLWH [103]. Post-mortem studies indicate that astrocytes, parenchymal microglia, and perivascular macrophages contain latent viral DNA prior to neurological symptoms or immunodeficiency [104]. Intriguingly, unintegrated HIV-1 long-terminal repeat (LTR) DNA is found in multinucleated giant cells in HAD patient brains, along with high levels of HIV proteins, suggesting productive infection despite low levels of DNA integration [105]. These clinical findings support that HIV latency, chronic HIV replication, and proinflammatory activation, processes that cannabis use appears to counter based on peripheral clinical observations, may be important targets for the modification of HIV pathology in the CNS. Indeed, studies in SIV-infected macaques show that Δ9-THC alters the expression of microRNAs that govern pathways associated with neurotrophin signaling, MAPK signaling, the cell cycle, and immune response [106]. In addition, BBB integrity, which is disrupted in HIV infection, is protected in PLWH who use cannabis frequently, as measured by the CSF-to-serum albumin ratio and CSF levels of soluble urokinase plasminogen activator [107]. 

While these reports begin to tease apart the cellular and molecular mechanisms that explain clinically observed interactions between cannabinoids and HIV, a deeper understanding is necessary in order to better predict the effects of cannabis abuse and identify possible cannabinoid-based therapeutic interventions in HAND. Basic in vitro and in vivo studies dedicated to this purpose allow for separation of cannabinoid receptor-specific and cell-type-specific cannabinoid actions, thus improving both our knowledge of the underlying biology and honing the specificity of novel therapeutic approaches.

## 3. Cannabinoids × HIV: Receptor- and Cell-Specific Mechanisms in Preclinical Models

### 3.1. CB_1_-Mediated Effects on HIV Neuropathology

Through basic research, the endoCB system has emerged as one of the key regulatory mechanisms in the brain, controlling multiple events such as mood, pain perception, learning, and memory, among others [108,109]. Evidence suggests that endoCBs play an important role in neurological disease and injury. They are thought to be neuroprotective during traumatic brain injury (TBI) and may be part of the brain’s natural compensatory repair mechanism during neurodegeneration [110,111,112]. The general contribution of CB_1_ activity to CNS disease is unclear, as data have shown that CB_1_ expression, which is found primarily on cortical pyramidal neurons [113], can increase, decrease, or remain stable in disease, depending on the brain region involved [59,114,115]. This emphasizes the need to contextualize regionality when assessing changes in receptor expression and activity. For example, in the presence of HIV-1 Tat, CB_1_ receptor expression is increased in prefrontal cortical neurons. In contrast, Tat-treated glioma cells express less CB_1_ along with inhibition of AEA reuptake and degradation, which could further downregulate CB_1_ receptor expression [116]. Therefore, the same HIV insult can lead to divergent effects on CB_1_ receptor expression in different models. Despite the conflicting literature on changes in CB_1_ and its expression levels in disease, it appears that its activity plays a key role in modulating synaptic function, especially in the context of neurodegenerative disorders.

The effects of CB_1_ activation on synaptic networks are complex. Different CB_1_ agonists display different voltage- and frequency-specific effects on EPSCs, and THC itself can act as an agonist or antagonist of CB_1_ at different voltages [117]. Voltage-sensitive coupling of CB_1_ to voltage-gated calcium channels facilitates these state-dependent actions of THC, suggesting that in addition to consideration of specific brain regions and neuronal subtypes, baseline electrophysiological conditions must be accounted for in analyses of CB_1_ pharmacology.

HIV-1 trans activator of transcription (Tat)-mediated increases in glutamatergic neurotransmission have been demonstrated in previous in vitro studies [118,119,120,121] and are suggested to be mediated via Tat’s actions on NMDA receptors [122] as well as non-NMDA, excitatory amino acid receptors [123]. Further, the excitotoxic effects of Tat also coincided with calcium influx, oxidative stress, and substantial neuronal damage [124]. Tat-induced increases in excitability, with enhancement of sEPSC frequency but no effect on amplitude, have been reported recently in vitro [121] and suggest a presynaptic stimulatory effect on glutamate release [118]. Interestingly, CB_1_ stimulation has been shown to limit glutamate-mediated synaptic excitation [125]. The involvement of CB_1_ located on glutamatergic terminals is critical for the neuroprotective effects of endoCBs in counteracting excitotoxicity [126]. endoCBs are secreted perisynaptically to engage with presynaptic CB_1_ and inhibit excitatory transmission in response to excess release of glutamate, thus buffering against the excitotoxic effects of NMDAR activity in the postsynaptic neuron [126]. Experimental evidence also demonstrates that Tat impairs CB_1_-mediated presynaptic inhibition at excitatory but not inhibitory terminals. Many in vitro models demonstrate a neuroprotective role for cannabinoids in the context of HIV excititoxicity [121,127,128,129,130]. The endoCB system prevents HIV-1-induced cytotoxicity in rat glioma cells [116]. These effects are reversed by CB_1_, but not CB_2_, antagonists [116]. Similarly, HIV Tat increases intracellular calcium in mouse prefontal cortex neurons, but prior treatment with anandamide (AEA) leads to significantly less neuronal damage [121]. This illustrates the primary role for CB_1_ in the context of HAND, as a local regulator of hyperactivity. HIV-1 Tat also reduces miniature inhibitory postsynaptic currents (mIPSCs) in mouse prefrontal cortex slices [131]. Application of WIN55,212-2 and AEA reduces the frequency, but not the amplitude, of mIPSCs and occludes further suppression of inhibitory signaling by Tat [131]. This selective attenuation of endoCB signaling may unbalance network excitability, with potentially significant effects on symptoms associated with HAND, the progression of neurological disease in people living with HIV, and the sensitivity of HIV-positive individuals to exogenous cannabinoids. 

Various studies have shown that CB_1_ activity mediates the neuroprotective effects ofendoCBs against both excitotoxic damage and inflammation. For example, 2-AG prevented increased expression of COX2, a proinflammatory enzyme, in neurons following inflammatory and excitotoxic stimuli via CB_1_ [132]. However, it is notable that even though activation of CB_1_ is known to be neuroprotective, substantial evidence suggests CB_1_ agonism, via a selective agonist or THC, can result in adverse psychoactive side effects such as sensorimotor, affective, and cognitive disturbances [133]. These side effects limit the feasibility of using cannabis or THC analogues as designed HAND therapies. CB_2_ agonists, however, are not psychoactive, opening the door for effective immunomodulation without CB_2_-specific synthetic cannabinoids.

### 3.2. CB_2_-Mediated Effects on HIV Neuropathology

#### 3.2.1. Viral Replication and Immunogenicity

Receptor-specific, cell-level experiments provide an ideal model for understanding the clinical observations of reduced HIV viral load in cannabis users. HIV’s primary target, CD4^+^ T cells, also express co-receptors, including CXCR4. CB_2_ agonists can suppress CXCR4-tropic HIV replication by disrupting downstream CXCR4 signaling pathways [134]. Similar effects are achieved by a variety of cannabinoids [135]. Denbinobin, a phytocannabinoid, inhibits HIV replication in T cells by disrupting TNF-α-induced NF-κB binding to the HIV-1 LTR [136], and N-arachidonoyldopamine (NADA), an unconventional endoCB, inhibits HIV replication in T cells by NF-κB-driven transcription of integrated viral genes [137]. These findings suggest cannabinoid interference in HIV replication occurs by disrupting both entry and viral gene transcription.

In the brain, HIV replicates primarily in macrophages and microglia. This is mediated primarily by the co-receptor CCR5, which facilitates similarly effective HIV tropism in both microglia and macrophages [138,139]. In macrophages and microglia, HIV infection increases CB_2_ expression [140]. WIN55,212-2 inhibits HIV replication and CCR5 expression in microglia, primarily via action on CB_2_ [141]. Additionally, exposure of differentiating monocytes to Δ9-THC suppresses HIV infection and virulence in resultant macrophages via CB_2_, consistent with monocytes expressing CB_2_ but little, if any, CB_1_ [142,143]. This was attributed to a number of factors, including downregulation of HIV cell surface receptors CD4, CCR5, and CXCR4 and increased expression of viral restriction genes [144]. Other reports suggest CB_2_ agonists suppress HIV replication in macrophages without altering surface receptor expression, primarily by suppressing HIV *pol* expression [135,140]. HIV infection in monocyte-derived dendritic cells is also attenuated by cannabinoids [145]. Thus, cannabinoid disruption of HIV replication in monocyte-derived cells mirrors that in T lymphocytes with distinct observation of surface receptor and transcription-based inhibition.

Another important consideration is how cannabinoids effect the specific immune response to HIV. Δ9-THC can enhance HIV gp120-antigen immune response [146]. Chen et al. also observed differential HIV gp120-antigen responses in T cells are dependent on the dose of cannabinoids and subsequent changes in the amount of intracellular calcium [147]. Similar findings were seen in mouse models, where perinatal Δ9-THC exposure reduced T cell response to HIV envelope antigens [148]. Intriguingly, in HIV-infected, severe combined immunodeficient (SCID) mice with implanted human peripheral blood leukocytes (PBLs), Δ9-THC suppresses CD4^+^ cell counts and increases both circulating viral load and the proportion of infected PBLs [149]. Thus, cannabinoids may restrict the initial immune response to HIV, leading to a greater number of initially infected cells and then, paradoxically, suppress its replication. These findings are consistent in both monocytes and lymphocytes, in the periphery and in the brain, and are primarily associated with altered HIV surface receptor expression and impaired transcription of the integrated viral genome. Further work is necessary to determine whether cannabinoid administration can lead to effective viral elimination or simply supports long-term latency.

#### 3.2.2. Inflammation and Immune Response

Extensive research identifies CB_2_ agonism, whether by endo- or exocannabinoids, as a modulator of inflammation and immune response. Endogenously, AEA acts through CB_2_ to inhibit IL-12 and IL-23 production in microglia and enhance IL-10 production by suppression of NF-κB, highlighting the importance of both downregulating proinflammatory pathways and stimulating anti-inflammatory cytokine release [150,151]. Gabrielli et al. report that microglia release active AEA on microcellular vesicles, providing a pathway for remote regulation of activation [152]. Exogenously activating CB_2_ suppresses a number of proinflammatory pathways in macrophages and microglia, including TNF-α, IFN-γ, and nitric oxide [153,154,155]. These effects also alter the pathology of nearby neurons, as CB_2_-mediated suppression of microglial nitric oxide was shown to reduce neuropathic pain [156]. Astrocytes also serve as targets for CB_2_ immunomodulation. Δ9-THC- and CB_2_-specific agonist treatment of monocytes suppresses IL-1β release, reducing secretion of monocyte chemoattractant protein 1 and IL-6 from co-cultured astrocytes [157]. WIN55,212-2 signaling via CB_2_ also directly inhibits IL-1β-induced MAPK phosphorylation in astrocytes, reducing CX3CL1 release [158]. In addition to our general understanding of CB_2_ as a regulator of microglia and astrocyte activation, considerable research supports its role in HIV-specific neuroinflammation.

Individual HIV molecular components activate glia. HIV-1 Tat increases microglial proinflammatory cytokine gene expression (including TNF-α, IL-1β, IL-6, RANTES, and MP-1α) and alters mitochondrial metabolism, resulting in impaired mitophagy [159,160]. These changes have also been observed in HAND patient brain tissue [161]. In Mueller glia, endoCBs can disrupt Tat-induced activation by disrupting NF-κB-driven inflammatory gene expression [162]. CB_2_ activation also inhibits macrophage and microglial migration toward Tat, and the chemoattractant RANTES [163,164,165], which may be explained by a high level of CB_2_ expression at the leading edge of microglial lamellipodia [166]. This expression pattern may also underly the observation that, in a murine HIVE model where infected human MDMs are injected into the brains of humanized mice, the CB_2_-specific inverse agonist, Gp1a, reduced infected human macrophage and human lymphocyte infiltration into the brain [167]. 

One key mechanism of HIV-induced neuroinflammation is activation of the NLRP3 inflammasome [168,169,170,171,172]. This process, triggered by pathogen and damage-associated signals, results in priming of NF-κB-driven genes that drive a positive feedback loop of proinflammatory IL-1β and IL-18 secretion [173]. This process has been observed in microglia in HIV^+^ post-mortem tissue and in human microglia and macrophage cultures [168]. HIV single-stranded RNA, Tat, and viral protein R are all independently capable of activating the NLRP3 inflammasome in microglia, and countering this activation can rescue microglia-mediated neurotoxicity [174,175,176]. Recent reports have found that CB_2_ activation stimulates autophagy and suppresses NLRP3 inflammasome activation in multiple inflammatory contexts, including in activated microglia in experimental autoimmune encephalitis [177]. Thus, CB_2_ activation may be an effective means to counter HIV-driven neuroinflammation, especially NLRP3 inflammasome activation, and prevent neuronal damage.

#### 3.2.3. Neurotoxicity

Although CB_2_ is not expressed on neurons, CB_2_ agonism is protective in a number of neuroHIV models, with that protection likely mediated by cannabinoid action on nearby glia. For instance, non-specific cannabinoids act via CB_2_ to protect dopaminergic neurons from HIV gp120-induced, microglia-mediated toxicity, and CB_2_ agonists prevent hippocampal neurotoxicity and stimulate neurogenesis in response to astrocytic gp120 expression [178,179]. These studies suggest that the anti-inflammatory effects observed in microglia, macrophages, and astrocytes can prevent non-cell autonomous neurotoxicity.

CB_2_ agonism also offers protection from peripheral neurological sequelae of HIV infection. CB_2_ knockout potentiates the development of neuropathic pain, while overexpression attenuates via increased CB_2_ in bone-marrow-derived cells [180]. CB_2_ agonism is protective in both HIV- and ART-induced neuropathic pain [181,182,183,184]. These findings corroborate and expand upon the frequent use of cannabis to treat pain in both ART-treated and ART-untreated HIV^+^ populations.

### 3.3. BBB Integrity

Considering the clinical evidence supporting BBB disruption in both HIVE and in chronic, ART-treated HIV infection, numerous reports have focused on the cellular and molecular pathways that govern brain microvascular endothelial cell (BMECs) dysfunction and transmigration of infected cells across the barrier [185,186]. BMECs cultured with HIV-infected MDMs show upregulated inflammatory gene pathways, including TNF-α, interferon, STAT1, and NF-κB-associated transcripts [187,188]. In addition to proinflammatory cytokine secretion, HIV-infected macrophages, microglia, and astrocytes release β-chemokines that drive monocyte migration across the BBB, thereby disrupting CNS immune privilege and facilitating easier entry of infected monocytes [189]. However, a robust interferon response in BBB endothelial cells can suppress HIV replication in nearby macrophages, suggesting that restoring BBB health and function may provide broader benefits [190].

Cannabinoids, particularly CB_2_ agonists, have demonstrated efficacy in countering inflammatory BBB disruption. CB_2_ stimulation in BMECs and co-cultured macrophages reverses altered gene expression driven by TNF-α and LPS activation [191]. CB_2_ agonists also disrupt monocyte adhesion to BBB extracellular matrix proteins and migration across the BBB by suppressing Rac1 and RhoA, which may prevent infiltration of infected monocytes [192,193]. Similarly, CB_2_ agonists also suppress LPS-driven leukocyte adhesion and prevent leakiness in the BBB [194]. In addition to cell motility and adhesion, cannabinoids can regulate the maintenance of BBB tight junctions, which are responsible for cellular exclusion and maintenance of chemical gradients. Lu et al. observed that HIV-1 Gp120-induced loss of tight junction proteins can be rescued by administration of cannabinoid agonists both in vitro and in vivo in a CB_1_-dependent manner [195]. These results suggest that cannabinoid agonists can provide protection of BBB integrity, preventing both infiltration and spread of HIV infection into the CNS and regulating CNS exposure to both central and peripheral inflammatory signaling.

### 3.4. Other Putative Cannabinoid Receptors

Although limited, a few studies of alternative cannabinoid receptors suggest they may also be viable targets for manipulation of neuroinflammation. The most well-characterized non-canonical cannabinoid receptor, GPR55, is differentially expressed in microglia activated by stimuli such as LPS and IFNγ in a stimulus-specific manner. This stimulus-specific expression mirrors alterations in CB_2_ expression via the same stimuli [196]. GPR55-specific antagonists reduce LPS-induced COX-2 activation and PGE_2_ release in microglia [197], suggesting that the upregulation in response to activation may be counter-regulatory. Growing evidence suggests GPR55 activation stimulates neural stem cell proliferation and differentiation in vitro and in vivo and is protective against IL-1β- and LPS-associated inflammatory disruption of neurogenesis [198,199]. In activated microglia, the putative cannabinoid receptor GPR18 forms a heteroreceptor with CB_2_, which is suggested to facilitate negative cross talk between the two [200]. These early reports suggest that expression and localization of alternative cannabinoid receptor can be altered in neuroinflammatory states such as HIV infection and that the effects of endo- and exocannabinoids on these receptors may serve immunomodulatory functions, either through direct action on receptor activation or through coupling to canonical receptors.

### 3.5. Endocannabinoid Enzyme Modification

Leveraging the endoCB system to study and counter mechanisms of HAND extends beyond targeting cannabinoid receptors. Manipulating the expression and function of synthesizing and hydrolytic enzymes can alter the availability and effect of endoCB synthesis and also affect the pharmacokinetics of exocannabinoids. In a mouse model of antiretroviral, nucleoside reverse transcriptase inhibitor-induced neuropathic pain, zalcitabine administration downregulated transcription of Faah and Mgll, establishing the possibility that alterations in these enzymes occur following ART [201] and that these enzymes may be targets for neuropathic pain intervention. Accordingly, pharmacologic inhibition of FAAH, which degrades AEA, counters HIV gp120-induced allodynia in a CB_1_- and CB_2_-dependent manner [202]. A separate study identified that pharmacologically inhibiting or knocking down FAAH reduces LPS-associated increases in PGE_2_ and proinflammatory cytokines. FAAH knockdown, but not pharmacological inhibition, also increases anti-inflammatory cytokine gene expression [203], suggesting that the efficacy of FAAH inhibition in treating allodynia is associated with endoCB immunomodulation.

Inhibition of FAAH also reduces Tat-mediated increases in intracellular calcium, neuronal cell death, and dendritic degeneration in mouse neuronal cells [127]. FAAH knockout is also impactful in GFAP-driven, gp120-expressing mice, resulting in higher levels of endoCBs and enhanced neurogenesis [130]. These mice also exhibit less astrogliosis and gliogenesis in the hippocampus. In addition, gp120 administered directly to the CNS of adult rats activates neocortical FAAH and enhanced AEA degradation, which leads to neuronal apoptosis; inhibition of FAAH reduces gp120-induced apoptosis [204], further establishing a protective role for preventing endoCB degradation.

Observations of MAGL inhibition echo this concept. Deletion of MAGL from astrocytes reduces LPS-induced inflammation in a CB_1_-independent manner [205], and pharmacological inhibition of MAGL reduces IL-1β secretion and PGE_2_ production in a CB_2_-dependent manner. This inhibition was protective in a primary rat hippocampal model of HIV gp120 neurotoxicity [128]. The CNS effects of MAGL inhibition are cell type dependent, as a study found that inhibition of MAGL can actually enhance spontaneous neurotoxicity in primary neuronal cultures. However, in mixed neuro-glial cultures, inhibition of MAGL only increased toxicity when CB_2_ receptors were antagonized, suggesting that glia compensate for MAGL loss in a CB_2_-dependent manner [206]. In future HAND research, measuring endoCB processing enzymes and interrogating their function will enhance our understanding of the ECS in HIV and might serve as targets for anti-inflammatory therapeutics.

## 4. Human iPSC Modeling of HIV in the CNS

The preclinical studies discussed above have elucidated several possible interactions between HIV and cannabinoids in the CNS, but many lack the biological context necessary to interpret their findings in relationship to clinical observations or translate their conclusions into therapeutic interventions. While small-animal models allow for investigations of disease progression and assessments of complex behavior that are unattainable in vitro, the human-specific nature of HIV greatly impedes translatability. Animal exposure to (or transgenic expression of) single molecular components of HIV does not incorporate the full range of known HIV toxins or allow for infection/replication efficiency to act as a modulator of pathology, further decreasing generalizability [207]. Recent advances in humanization and recombinant challenge viruses have increased the number and sophistication of available models; however, the associated immune system disruption and non-canonical virus replication severely limit the study of the immunomodulator and replication inhibitor roles of cannabinoids.

**Figure 1:**
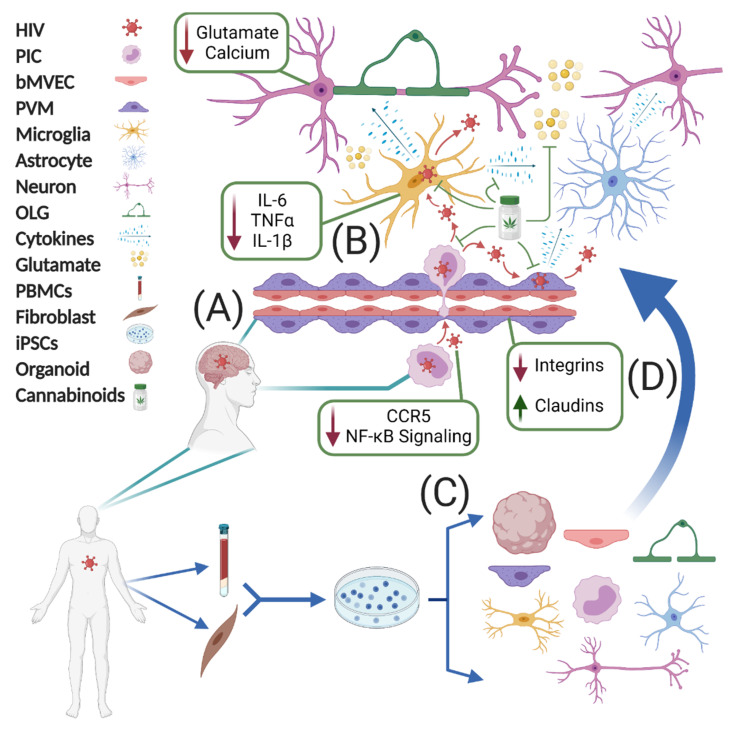
Human iPSCs can model the multicellular actions of cannabinoids in neuroHIV. (**A**) HIV crosses the BBB, infecting macrophages and microglia. This results in a cascade of proinflammatory activation and excitotoxicity that manifests as HAND. (**B**) Cannabinoids are uniquely suited to counter the two primary HIV-associated neurotoxic mechanisms by modulating neurotransmission and suppressing inflammation. Green boxes indicate molecular mechanisms through which cannabinoids counter HIV neuropathology at various sites of action. (**C**) Recent advances in iPSC technology allow for both 2D and 3D human models of many CNS cell types. (**D**) Combining iPSC-derived CNS cells and exposing them to HIV and cannabinoids provides a platform to study common HAND comorbidities in a holistic, human context. Abbreviations: HIV, human immunodeficiency virus-1; HAND, HIV-associated neurocognitive disorders; PIC, peripheral immune cell; bMVEC, brain microvascular endothelial cell; PVM, perivascular macrophage; OLG, oligodendrocyte; PBMC, peripheral blood mononuclear cell; iPSC, induced pluripotent stem cell. Created with BioRender.com.

In contrast, human in vitro models allow for examination of unadulterated HIV biology. Still, the in vitro studies cited here frequently suffer from the difficulty of modeling CNS cell types. Due to the paucity of live brain biopsy tissue, primary culture of human CNS cells is challenging at best and completely infeasible at the scale necessary to perform drug screens or examine tripartite interactions. In addition, immortalized cell lines (especially neuronal) fail to replicate primary functions of the cells they model. Lastly, while monocultures are useful for determining cell-specific effects of a given treatment, they fail to recapitulate the non-cell autonomous nature of HAND neurotoxicity. Thus, the advancement of HIV and cannabinoid research requires scalable human models of interacting CNS cell types (Figure 1).

Over the past decade, there have been considerable advances in the use of human indued pluripotent stem cells (hiPSC) to model specific CNS cell types and neuronal subtypes [208]. Transcription factors or small molecules differentiate these human cells into a state morphologically, transcriptionally, and functionally similar to their primary counterparts, including neurons, astrocytes, microglia, macrophages, and endothelial cells [209]. Recently, our lab published data demonstrating a forebrain hiPSC triculture system with neurons, astrocytes, and microglia [9]. This method rapidly produces microglia-like cells (iMg) that express multiple classical markers that can be productively infected with HIV and respond to ART. While this system is a first step in the generation of multicellular CNS cultures, three-dimensional (3D) models that incorporate brain macrostructures and replicate the blood–brain barrier will incorporate several advantages of in vivo models and allow for examination of the effects of peripherally administered HIV, cannabinoids, and ART on the brain. To this end, integrating iMgs into existing 3D forebrain organoid models [210,211,212] offers an opportunity to observe infected human microglia in spatial context with neurons, other glia, and larger brain structures. Further co-culture or organoid designs that use 3D-printed vasculature, microfluidic devices, or permeable supports alongside human BMEC cells offer the potential to overcome one of the most significant limitations of in vitro modeling: the lack of a functional BBB [213,214,215]. Unfortunately, current in vitro models cannot replicate oligodendrocytes with functional myelination, which restricts our understanding of HIV and ART white matter pathologies. While differentiating and maintaining iPSC models can be expensive and time consuming, increased differentiation efficiency and more rapid differentiation offer the possibility of high-throughput iPSC modeling, which is necessary and, excitingly, sufficient to delineate the complex three-way cross talk between HIV, cannabinoids, and ART that is present in many people living with HIV and associated neuropathology.

## 5. Conclusions

Cannabinoids, be they endocannabinoids, cannabis derived, or synthetic therapeutics, are increasingly common and potent modulators of neurotransmitter release and neuroinflammation. The studies summarized here identify these biological processes as key components of HIV neuropathogenesis and highlight the necessity of considering the impact of cannabinoid exposure both in clinical studies and in the development of clinically relevant models. Beyond their role as a prevalent confounding variable, the diverse pharmacology of cannabinoids suggests that while they may initiate and exacerbate neurocognitive deficits, their neuro- and immuno-modulatory properties can be neuroprotective. Understanding the cell- and receptor-specific effects of cannabinoids, as well as their downstream mechanisms of action, offers an opportunity to isolate and leverage their neuroprotective properties to treat neuroinflammatory conditions with brain-permeable compounds. By applying the knowledge synthesized above with targeted pharmacology in modern human cell models, we can learn how to correct for cannabinoids’ confounds, separate their actions that cause neurocognitive disruption, and, perhaps, leverage their inhibitory functions in the search for a cure.

## Data Availability

Not applicable.
